# Hypothyroxinemia in sick term neonates and its risk factors in an extramural neonatal intensive care unit: a prospective cohort study

**DOI:** 10.20945/2359-3997000000500

**Published:** 2022-06-02

**Authors:** Ruchi Rai, D. K. Singh, Bhanu Kiran Bhakhri

**Affiliations:** 1 Super Speciality Pediatric Hospital Postgraduate Teaching Institute Department of Neonatology (Maternal Reproductive Health) Noida UP India Department of Neonatology (Maternal Reproductive Health), Super Speciality Pediatric Hospital and Postgraduate Teaching Institute, Noida, UP, India; 2 Super Speciality Pediatric Hospital Postgraduate Teaching Institute Department of Pediatrics Noida UP India Department of Pediatrics, Super Speciality Pediatric Hospital and Postgraduate Teaching Institute, Noida, UP, India

**Keywords:** Euthyroid sick syndrome, neonatal intensive care unit, term newborns, thyroid disturbances

## Abstract

**Objective:**

Thyroid functions in the sick newborns may be altered in the first week of life. Transient hypothyroxinemia has been reported in these babies, which could be due to the immaturity of the hypothalamic-pituitary-thyroid axis or to acute illness. We conducted this study to estimate the incidence of hypothyroxinemia and determine its risk factors in sick term newborns.

**Materials and methods:**

We analyzed free T4 (FT4) and thyroid-stimulating hormone (TSH) levels in sick term neonates (≤7 days of life) admitted to the neonatal intensive care unit. FT4 and TSH levels were estimated in the first week of life in all the enrolled neonates (N = 98) and then repeated at 14-21 days of life in 46 babies. Risk analysis was conducted using univariate and multivariate logistic regression, and numerical data was compared using the Mann-Whitney U test and t-test.

**Results:**

Hypothyroxinemia was seen in 10 (10.2%) of the admitted term babies. Male gender, vaginal delivery, presence of hypoxic ischemic encephalopathy, and need for mechanical ventilation (>24 hours) were identified as risk factors. There was a significant negative linear correlation between FT4 level in the first week of life and duration of hospital stay.

**Conclusion:**

Hypothyroxinemia is common in sick term neonates.

## INTRODUCTION

Disturbances in thyroid function in sick and preterm babies are common (1-3). Morbidity during hospital stay, drug usage, and abnormalities in transition to extrauterine life all affect newborns’ thyroid hormone homeostasis in the immediate neonatal period. These neonates can have hypothyroxinemia (HT), euthyroid sick syndrome (ESS), and congenital hypothyroidism (CH) with or without delayed elevation of thyroid-stimulating hormone (TSH). Thyroid dysfunction in preterm neonates has been studied extensively in the past, but studies in sick term babies are limited (3,4). Transient hypothyroxinemia of prematurity (THOP) is a well-understood entity, and many studies have highlighted the issue of preterm babies’ thyroid dysfunction and its impact on long-term neurological outcomes, but similar studies in term babies are lacking (5,6). Sick term babies also experience abnormalities in thyroid function.

Some studies have tried to evaluate the role of thyroid function as a prognostic marker for severity of disease and poor outcome in term neonates (7,8). Low thyroxine (also known as T4) levels have been associated with increased mortality, skull abnormalities in ultrasounds, and the need for rescue interventions in ventilated neonates (4,9).

ESS, or non-thyroidal illness syndrome, is a known entity in older children and adults but is not yet very well-defined in neonates (10). It is caused by increased levels of circulating cytokines and other inflammatory mediators resulting from non-thyroidal illnesses. This causes decreased T3 levels and may be associated with decreased T4, free T4 (FT4), and TSH levels in severe illness (11,12).

CH with delayed TSH elevation has been reported not only in very low birth weight, extremely low birth weight, and preterm babies but also sick term babies admitted to the neonatal intensive care unit (NICU) (5,13,14). This condition is defined by elevated TSH at the second neonatal screening after a normal first screening, irrespective of the T4 level (13). The European Society for Pediatric Endocrinology and the Indian Society for Pediatric and Adolescent Endocrinology recommend that a repeat screening for such high-risk babies should be done at 2-4 weeks, so that cases of CH with delayed TSH elevation are not missed (15-17).

There is a paucity of studies on thyroid function abnormalities in sick term babies, especially from India. Therefore, we conducted this study to estimate the incidence of HT in sick term newborns and determine its risk factors.

## MATERIALS AND METHODS

This was a prospective cohort study conducted in the NICU of a tertiary-level teaching institute. The 20-bed level III unit caters to extramural babies. The study was done over a period of 1 year, from April 2019 to March 2020. The study was approved by the institutional ethics committee of SSPHPGTI (IEC Ref code: 2019-02-IM-01). Informed consent was taken from the mother and/or father of each baby in the study.

The **primary objective** of the study was to estimate the incidence of HT in sick term babies. The **secondary objective** was to determine the risk factors for HT in term babies admitted to the NICU.

### Inclusion criteria

Neonates admitted to the NICU were eligible for inclusion in the study if they fulfilled the following criteria:

1. Gestational age ≥ 37 weeks *and*
2. Admitted at ≤ 7 days of life.

### Exclusion criteria

Neonates admitted to the NICU were excluded from the study if they fulfilled the following criteria:

1. Admitted only for observation *or*
2. discharged within 72 hours of admission.

All newborns admitted to the NICU over the period of 1 year who fulfilled the inclusion criteria were enrolled in the study. Their details were entered in a pro forma, which gathered demographic data like birth weight, gestational age, and gender, and medical details like duration of hospital stay, morbidities, and administration of dopamine within 72 hours of admission. The newborns were managed according to standard treatment guidelines. Hypoxic ischemic encephalopathy (HIE) was classified per Levene staging (18). For all babies admitted within 24 hours of birth, 2 ml of venous sample (**Sample I**) for FT4 and TSH was sent at 48-72 hours of life; for babies admitted after 48 hours of birth, the sample was submitted on the day of admission. A repeat venous sample (**Sample II**) was sent for FT4 and TSH at 14-21 days of age in babies with a stay ≥ 7 days. The first samples were FT4 (1) and TSH (1) and the repeat samples were FT4 (2) and TSH (2). The samples were analyzed using an enzyme-linked automated fluorescent immune assay technique (VIDAS, bioMerieux). HT was defined as FT4 (1) levels < 12 pmol/L (16,17). HT was considered transient if the FT4 values normalized in the repeat estimation done at 14-21 days of life (FT4 [2)).

### Statistical analysis

Statistical analysis was conducted with Epi Info 7 and SPSS 27. The normally distributed numerical data was analyzed by t-test. The Mann-Whitney U test was used to compare the numerical data that was not normally distributed. A p-value of <0.05 was considered significant. Pearson's correlation coefficient was used to assess the correlation between continuous variables. Univariate and multivariate logistic regression was used to study the association.

## RESULTS

**Figure 1:**
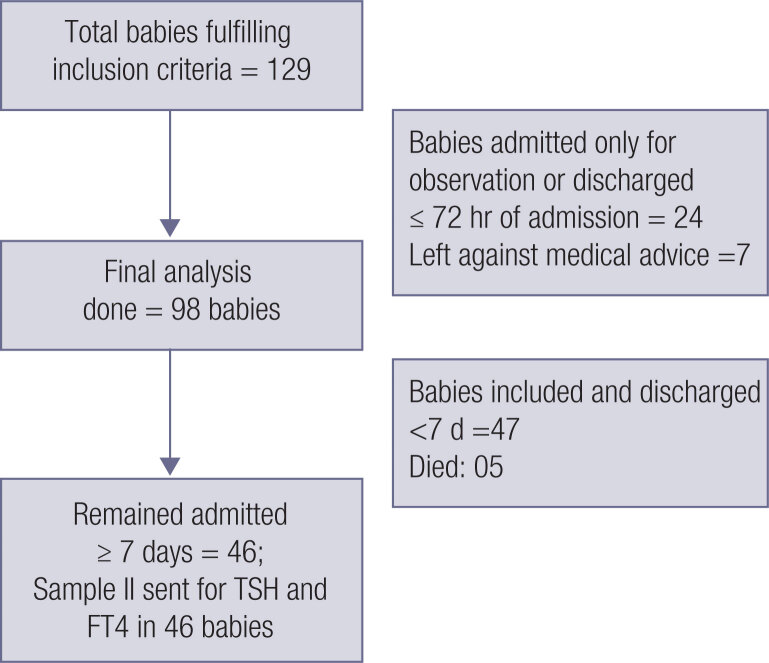
Flow chart for the study plan.

After the study duration of 1 year, a total of 98 babies were analyzed (Figure 1). The characteristics and morbidity pattern of the study population are described in Table 1. There was a significant negative linear correlation between FT4 (1) value and duration of hospital stay (coefficient = −0.28; p-value = 0.005), signifying that lower FT4 levels were significantly associated with longer hospital stays. The FT4 (1) levels were significantly lower in babies born by vaginal route, those who suffered from HIE (moderate or severe), babies needing mechanical ventilation (>24 hours) and those who needed dopamine infusion within 72 hours of admission (Table 2). Gender and sepsis did not affect the mean FT4 (1) level significantly.

**Table 1:** Characteristics of study population

Characteristics		N = 98 N (%)
Age at admission (d)		2.3 (1.6)^1^
M:F		2.4:1
Vaginal delivery		78 (79.5%)
Birth weight (g)		2,581 (492)^1^
Stay (d)		8.5 (5,14)^2^
Perinatal asphyxia		58 (59.2%)
HIE (moderate or severe)		54 (55.1%)
Sepsis		12 (12.2%)
Received dopamine		10 (10.2%)
Received MV		25 (25.5%)
Outcome		
	Discharge	93 (94.9%)
	Died	5 (5.1%)

1 Mean (standard deviation).

2 Median (inter quartile range).

MV: mechanical ventilation (>24 h); HIE: hypoxic ischemic encephalopathy.

**Table 2:** FT4 (1) and TSH (1) levels in the study population

		FT4 (1) (pmol/L) mean (SD)	p value	TSH (1) (mIU/L) median (IQR)	p value
Sex	M	19.8 (7.8)	0.05	2.9 (1.7,5.8)	0.69
	F	23 (5.7)		4.2 (2.2,7.5)	
MOD	Vaginal	19.7 (7.1)	<0.01^1^	2.9 (1.7,5.8)	0.25
	LSCS	24.89 (7)		4.35 (2.1,8.1)	
MV	Yes	16.81 (6.7)	<0.01^1^	2.84 (0.9,5.8)	0.29
	No	22.16 (7.1)		3.19 (1.9,6.6)	
HIE	Yes	17.3 (6.2)	<0.01^1^	2.9 (1.6,7.8)	0.99
	No	24 (6.9)		3.1 (1.9,6.1)	
Dopamine	Yes	15.45 (4.3)	<0.01^1^	2.45 (0.5,3.1)	<0.02^1^
	No	21.4 (7.4)		3.2 (1.9,7.4)	

1 p value significant.

SD: standard deviation; IQR: inter quartile range; MOD: mode of delivery; LSCS: lower segment caesarian section; MV: mechanical ventilation (>24 h); HIE: hypoxic ischemic encephalopathy (moderate or severe).

Hypothyroxinemia was seen in 10 babies (10.2%; 95% confidence interval [5%, 18%]), all of whom were male and delivered vaginally. None of the babies with HT had elevated TSH (1), suggesting that it was not due to CH. Univariate logistic regression analysis showed that presence of moderate or severe HIE, need for mechanical ventilation (>24 hours), and dopamine administration were significantly associated with HT. In multivariate regression analysis, however, only HIE and need for mechanical ventilation (>24 hours) were found to be independently associated with HT (Table 3). Low FT4 (1) levels were not associated with increased mortality. Out of the 10 babies with HT, repeat estimation of FT4 and TSH was done at 2-3 weeks in 8 babies. A repeat estimation was not done for 2 of the babies with HT, as they were discharged within 7 days. The FT4 levels normalized in 6 of these babies, suggesting that HT was transient in the babies. The levels remained low in 2 babies.

**Table 3:** Univariate and multivariate logistic regression analysis

	OR	p value	Adjusted OR^1^	95% CI	p value
HIE	11.3	0.02^2^	9.7	1.1,83.2	0.03^2^
Stay ≥14 d	7.44	0.08	-	-	
MV	3.19	0.006^2^	6.44	1.4,28.6	0.014^2^
Dopamine	9.8	0.001^2^	1.59	0.5,4.4	0.37

1 p value significant.

2 multiple logistic regression.

OR: odds ratio; CI: confidence interval; HIE: hypoxic ischemic encephalopathy (moderate or severe); MV: mechanical ventilation (>24 h).

There was no correlation between TSH levels in the first week of life and duration of hospital stay. The TSH (1) levels were significantly lower in babies who received dopamine, but did not differ significantly among the study population according to gender, mode of delivery, presence of sepsis, need for mechanical ventilation (>24 hours), and presence of HIE. None of the babies had elevated TSH in the first week.

## DISCUSSION

We studied the FT4 and TSH levels in 98 sick term newborns in their first week of life. Repeat FT4 and TSH levels were checked in 46 babies at 14-21 days. HT was seen in 10 (10.2%) of the babies, among whom it was found to be transient in 6 (60%). HT was significantly associated with male gender, vaginal delivery, presence of HIE, and need for mechanical ventilation (>24 hours). There was a significant negative linear correlation between FT4 (1) value and duration of hospital stay.

Low thyroid hormones, T4, FT4, T3, and free T3 have been reported in sick newborns and have been associated with poor outcomes. Most studies report the abnormality in thyroid function as transient. Goldsmit and cols. studied the clinical significance of changes in thyroid hormone and TSH in critically ill term newborns (7). The babies had significantly lower T3 and T4 levels in comparison to the healthy newborns. Babies with low levels of T3 along with low T4 and TSH were found to have higher risk of mortality, with a risk ratio of 10.75. Chen also demonstrated lower T4 values in sick term babies than in healthy controls, although the TSH levels were not significantly different (8). In neonates with sepsis > 35 weeks of gestation, Das and cols. evaluated thyroid hormone levels in relation to outcomes (9). They concluded that the babies had lower mean serum T4 at admission than healthy neonates did, but that T3 and TSH were comparable in both groups. This study also found that T4 levels normalized after recovery from illness and that T3 and T4 were lower in neonates who expired. Our study also showed significantly lower FT4 levels in newborns born vaginally, needing mechanical ventilation (>24 hours) and dopamine infusion, and suffering from moderate or severe HIE. We did not find any association between FT4 levels and mortality. The FT4 level, however, had a significant negative correlation with the duration of stay. TSH levels were not significantly different across the study population. The mean standard deviation of TSH levels in the admitted term babies in our study was much lower than that reported in healthy controls in previous studies (4.33 [4.36] *vs.* 8.45 [1.70]; 19).

Perinatal asphyxia is one of the most common indications for NICU admission in term babies in developing countries; it accounted for 59% of all admissions in our study population. Perinatal asphyxia affects the cellular function of all organs and results in diminished oxidative phosphorylation and ATP production. Therefore, perinatal asphyxia also plays an important role in thyroid function. Past studies show that thyroid function is affected in newborns who suffer asphyxia. Tahirović evaluated the effect of birth asphyxia on perinatal thyroid function (20,21). They found that the mean values of FT4 in the cord blood of babies with Apgar scores < 6 and in babies with acidosis were significantly lower. Sak and cols. found mean FT4 and T4 levels in cord blood of babies with Apgar scores of < 4 at 1 and 5 minutes to be lower than in that of matched controls (22). Mean TSH values, on the other hand, were significantly higher. Similar results were found by other authors as well (23). In some studies, the cord blood levels of TSH, T3, T4, and FT4 in asphyxiated babies did not differ from those in normal babies, but the levels done at 18–24 hours of birth showed significantly lower values (19,24,25). This suggests a central hypothyroidism secondary to asphyxia. We found the FT4 levels to be significantly lower in babies with HIE; presence of HIE was also an independent risk factor for HT.

Other morbidities have also been studied for their role in thyroid function. Kadivar and cols. found that thyroxine levels were lower in babies with sepsis or jaundice, though the difference was not significant (1). We did not find any difference in TSH and FT4 levels in babies with sepsis. In a study by Lim and cols., term babies with HT required more intensive rescue interventions like need for extracorporeal membrane oxygenation (ECMO) and inhaled nitric oxide (4). Our study had similar results, as the babies who needed mechanical ventilation had significantly lower FT4 levels.

HT seen in sick babies could be a part of ESS. Whether thyroxine levels are markers or mediators of the clinical outcome is still not clear. Silva and cols. assessed the thyroid hormonal changes in newborns with sepsis and found that 60% of the babies with sepsis had ESS, which was characterized by low T3 or low T3 and T4; the levels normalized after sepsis was cured (10). Dagan and cols. reported thyroid function changes suggesting ESS in babies undergoing open-heart surgery for congenital heart disease. They also found significantly lower FT4 levels in babies on higher ionotropic support, those who had higher Pediatric Risk of Mortality (PRISM) score, and those needing longer duration of ventilation (26). We did not assess T3 levels in our study, as FT4 levels have also been reported to be low in ESS with more severe disease (11). We had 10 babies who had low FT4 (1) in the first week; though the levels normalized at 14-21 days in 6 babies, FT4 may take much longer to return to normal in some babies.

Dopamine has been associated with HT of prematurity and it significantly decreases FT4 levels in preterm babies (27,28). The mechanism of this effect of dopamine on thyroid is not well understood. As dopamine is administered to infants who are severely ill, severity of illness may be a confounding factor. Dopamine receptors are expressed in the thyroid, pituitary, and hypothalamus and have been shown to suppress anterior pituitary hormones (28,29). Dopamine administration may also directly affect the thyroid gland (27). We found mean FT4 (1) and TSH (1) levels to be significantly lower in babies who received dopamine within 72 hours of admission, but this was not found to be an independent risk factor for HT.

The strengths of the study are: i) it is a prospective study and ii) we screened the babies for both TSH and FT4 levels simultaneously, the ideal approach. Limitations include our exclusion of T3 levels, which could have helped us deepen our understanding of thyroid dysfunction in the participants.

In conclusion, thyroid function abnormalities in sick term babies must be studied widely and in greater detail for a full understanding of the problem. This will inform the formulation of guidelines regarding thyroid function screening and the need for thyroid hormone replacement in those found to have low levels. There are currently no recommendations addressing the replacement of thyroid hormones in such babies. The long-term neurodevelopmental outcomes of babies experiencing HT in the immediate neonatal period also constitute a topic of research in need of attention.
